# Ethyl Pyruvate Combats Human Leukemia Cells but Spares Normal Blood Cells

**DOI:** 10.1371/journal.pone.0161571

**Published:** 2016-08-31

**Authors:** Gerd Birkenmeier, Nasr Y. A. Hemdan, Susanne Kurz, Marina Bigl, Philipp Pieroh, Tewodros Debebe, Martin Buchold, Rene Thieme, Gunnar Wichmann, Faramarz Dehghani

**Affiliations:** 1 Institute of Biochemistry, Medical Faculty, University of Leipzig, Johannisallee 30, 04103 Leipzig, Germany; 2 Department of Orthopedics, Trauma and Plastic Surgery, University of Leipzig, Liebigstr. 20, 04103, Leipzig, Germany; 3 Institute of Medical Microbiology, Medical Faculty, University of Leipzig, Liebigstr. 21, 04103 Leipzig, Germany; 4 College of Medicine and Health Sciences, Bahir Dar University, Bahir Dar, Ethiopia; 5 University Medical Center, Department of Visceral, Transplantation, Thoracic and Vascular Surgery, University of Leipzig, Liebigstr. 19, 04103 Leipzig, Germany; 6 ENT-Research Lab, Department of Otolaryngology, Head and Neck Surgery, University Hospital Leipzig, Medical Faculty, University of Leipzig, Liebigstr. 21, 04103 Leipzig, Germany; 7 Department of Anatomy and Cell Biology, Martin Luther University Halle-Wittenberg, Grosse Steinstrasse 52, 06097 Halle (Saale), Germany; Professor, GERMANY

## Abstract

Ethyl pyruvate, a known ROS scavenger and anti-inflammatory drug was found to combat leukemia cells. Tumor cell killing was achieved by concerted action of necrosis/apoptosis induction, ATP depletion, and inhibition of glycolytic and para-glycolytic enzymes. Ethyl lactate was less harmful to leukemia cells but was found to arrest cell cycle in the G0/G1 phase. Both, ethyl pyruvate and ethyl lactate were identified as new inhibitors of GSK-3β. Despite the strong effect of ethyl pyruvate on leukemia cells, human cognate blood cells were only marginally affected. The data were compiled by immune blotting, flow cytometry, enzyme activity assay and gene array analysis. Our results inform new mechanisms of ethyl pyruvate-induced cell death, offering thereby a new treatment regime with a high therapeutic window for leukemic tumors.

## Introduction

Leukemia is one of the main causes of death in cancer patients. Although chemotherapy is most frequently used in leukemia treatment, it has been associated with many side effects such as systemic cytotoxicity and multi-drug resistance [1–3].To overcome such problems, various anti-cancer drugs have been applied in combination or given together with substances that increase sensitivity of leukemia cells to chemotherapy such as butyrate [4].

Ethyl pyruvate (EP) has attracted increasing interest in new treatment modalities of different diseases such as malignancies, inflammation and reperfusion syndrome [5–8]. The mechanism of action is still unsolved and a number of different targets are reckoned. Based on earlier work of Fink et al. [9] EP substituted pyruvate as a ROS scavenger and antioxidant in clinical reperfusion syndrome management. Neuroprotective effects of EP have also been demonstrated *in vitro* and animal studies related to stroke [10], Parkinson disease [11] and spinal cord injury [12]. In most studies, a protective role of EP in cells, tissue or organs has been described however cell toxicity has been found only in tumor cells so far. EP slowed tumor growth in xenografts by inhibition of tumor cell proliferation, migration and induction of apoptosis and cell cycle arrest [6]. In a hepatic tumor growth model, EP revealed a growth inhibiting effect via induction of apoptosis and amelioration of host inflammation [7].

Recently, we demonstrated EP as an inhibitor of glyoxalases (GLO). These enzymes are responsible for degradation of the cytotoxic methylglyoxal (MGO) [13]. This metabolite is preferentially formed aside of the glycolytic pathway through non-enzymatic degradation of triose phosphates. MGO is largely produced in cells exhibiting a high glycolytic throughput such as tumor cells [14]. Because MGO exerts cytotoxic effects by inducing apoptosis and modification of nucleic acids and proteins, inhibition of MGO degradation might be a promising way to inhibit growth of highly proliferating cells such as leukemia cells. This was the rationale to test EP for combating the tumor cell growth.

In the present study we demonstrate inhibition of acute and chronic leukemia cell growth by EP and ethyl lactate (EL) through induction of necrosis/apoptosis, ATP-depletion and the involvement of GLO1, pyruvate kinase (PK) and lactate dehydrogenase (LDH). We clearly provide evidence that these compounds show an exceptionally high capability for targeting highly proliferative leukemia cells without affecting normal cognate blood cells.

Our results suggest new mechanisms of EP-induced cell death and offering thereby a new treatment regime with a high therapeutic window for leukemia.

## Materials and Methods

### Ethics

Human blood was obtained from male healthy volunteers in the age of 30 to 40 years. All participants provide their written informed consent to participate in this study. The local ethic committee of the Faculty of Medicine of the University of Leipzig, Germany, approved this study in accordance to the ICH-GCP guidelines (reference number:057-2010-08032010.

### Reagents

RPMI-1640 medium, fetal calf serum (FCS) and trypan blue were purchased from Seromed (Berlin); anti-human GLO1 monoclonal antibody (mAb, #02–14) was from BioMac (Leipzig, Germany); cell proliferation WST-1 reagent from Roche; anti-human β-actin mAb was from Abgent (Hamburg); HRP-labeled goat anti-mouse Ab and Real Detection System Peroxidase/3,3'-diaminobenzidine (DAB) Rabbit/Mouse Kit from Dako (Hamburg); anti-human GAPDH (cat.no. 5174), anti-human phospho(Ser9)-glycogensynthasekinase-3β (anti-phospho GSK3β (Ser9) (cat.no. 9322), anti-human GSK-3β (cat.no. 9315), pan-phospho-β-catenin (Ser33/37/Thr41) (cat.no. 9561) antibodies from Cell Signaling; protease inhibitor cocktail, RNAse, EP, EL¸ annexin-V-fluoresceine isothiocyanate (FITC), propidium iodine (PI) and LDH-1 were obtained from SigmaAldrich (Taufkirchen); chemiluminescence detection kit from Boehringer (Mannheim); RT² Profiler™ PCR Array: Human WNT Signalling Pathway(Cat. No. PAHS-043F-2) from SA Bioscience (Hilden); plasmid *pCMV-GLUC* was obtained from Prolume Nanolight Inc. (Pinetop, AZ); TCF-Reporter Plasmid Kit from Millipore (Schwallbach); TransIT®-LT1 from Mirus Corporation (Madison) and *Gaussia* luciferase transfection kit and coelenterazine from PJK (Kleinbittersdorf).

### Cell line and cell culture

Cell lines used for this study are the monocytic acute leukemia cell line (THP-1, ATCC No. TIB-202), human myeloid leukemia cell line (CML cell K-562) (ATCC, CCL-243), prostate cancer cell lines LNCaP (ACC No. 256, DSMZ), DU-145 (ACC 261, DSMZ), PC-3 (CRL-1435, ATCC), and astrocytoma cell line 1321N1 (ECACC, 86030102). Cells were cultured at a density of 10^6^ /mL in RPMI 1640 medium, containing penicillin (100U/mL), streptomycin (100μg/mL), glutamine (2mM) and 10% FCS. Cultures were incubated in a moist atmosphere with 5% CO_2_ at 37°C.

### Preparation of PBMC

PBMCs were prepared by density gradient centrifugation of EDTA-blood samples obtained from healthy volunteers. Diluted blood was layered onto Ficoll-Paque^®^ and centrifuged at 1100×*g* for 30 min at room temperature. Cells were then aspirated from the interface and washed three times with PBS and finally suspended in RPMI 1640 medium (10% FCS).

### Assessment of cell death

THP-1 cells (2x10^5^ cells/mL) were seeded into 24-well culture plates and treated with EP or EL (1–20 mM) in RPMI 1640, 10% FCS. Cells were centrifuged at 200 x g for 5 min, washed in sterile PBS and re-suspended in 200 μl binding buffer composed of 10 mM Hepes, 140 mM NaCl, 2.5 mM CaCl_2_, pH 7.4. After addition of 2.5 μl annexin-V-FITC and 2.5 μl PI, cells were incubated for 15 min at room temperature. Isolated PBMCs were treated and labelled similarly. Cells were then centrifuged, washed twice and re-suspended in 200 μl binding buffer and analyzed. Results were presented as percentage of vital, apoptotic and necrotic cells of total cells counted using a FACSCalibur and CellQuestPro Software (BD Biosciences). Scattered plots were evaluated as follows: the percentage in the bottom left quadrants represent vital cells (annexin-V- and PI-negative), bottom right quadrants represent the early apoptotic cells (annexin-V-positive), the top right quadrants correspond to necrotic and late apoptotic cells (annexin-V- and PI-positive), the top left quadrants shows necrotic cells (PI-positive). In case of PBMCs only monocytes were gated and analysed as described above.

### Cell viability and Proliferation assays

Trypan blue exclusion test was performed to evaluate cell viability. Cell proliferation was assayed in 96-well plates (5000 cells/well) using the WST-1 assay.

### Colony forming unit assay

Six-well plates were prepared as describe elsewhere [15] using RPMI medium for growth of THP-1 and K562 cells (2.5x10^5^)/well. Prior to plating cells were incubated with EP for 24 hours, centrifuged and washed followed by mixing with the agar. Cultures were evolved for certain days and replenished every third day with fresh medium without drug. Colonies were read after staining with crystal violet solution. Experiments were conducted in triplicates.

### ATP measurement

ATP content was determined by means of the CellTiter-Glo® Assay (Promega, Madison, USA). Briefly, THP-1 cells were cultured in 96-well plates (5000 cells/well) in medium in the absence or presence of EP or EL, respectively. After 6 and 24 hours, cells were mixed with test reagent and luminescence was read according to the manufacturer’s instructions. Standard curve was prepared from ATP (1 μM to 10 nM) in medium.

### Cell cycle analysis

Following treating with EL, THP-1 cells were washed and re-suspended in 500 μL wash buffer (PBS, 2% FCS). Cells were then fixed by 70% ethanol overnight at 4°C, washed twice, and re-suspended in 200 μL RNAse A solution (1 mg/mL in PBS) and incubated for 30 min at 37°C, and followed by addition of 400 μL wash buffer and 20 μL PI solution (1 mg/mL). Cells were analyzed on a FACSCalibur.

### Analysis of phagocytic and burst activity of blood cells

Quantitative leukocyte phagocytosis and oxidative burst assays were performed using the Phagotest and Phagoburst kits (ORPEGEN Pharma, Heidelberg) according the manufacturer’s instructions. Oxidative burst or phagocytosis was monitored by observing the FL1 channel mean fluorescence intensity (MFI) of monocytes and granulocytes separately.

### Immunoblotting

Cytosolic protein extracts of cells were prepared as previously described [16]. Protein content was determined by Bradford [17]. The protein amount of 10–40 μg were separated by SDS-pore gradient polyacrylamide gel electrophoresis (4–20%) under reducing conditions, blotted onto cellulose nitrate membranes and labelled by anti-human GLO1 mAb (1:1000), anti-human GSK-3β (1:1000) and anti-human P-Ser9-GSK-3β (1:1000); anti-pan-phospho-β-catenin (1:1000) Ab, respectively, in combination with goat anti-mouse Ig-HRP (1:1000). Rabbit anti-β-actin Ig (1:2000) in conjunction with HRP-labeled goat anti-rabbit Ig were used to detect β-actin. Band visualization was performed by chemiluminescence detection and relative band intensities of Western blots were analysed by E.A.S.Y. Win 32 software (Herolab, Wiesloch, Germany).

### Enzyme activity measurement

GLO1 activity was measured as described previously [18]. The glycolytic enzymes were analyzed as described previously [19]. LDH-1 activity was measured at increasing concentrations of EP (1 to 20 mM) and Vmax and Km was calculated by GraphPad Prism software.

### Isolation and quality control of RNA

Total cellular RNA of 2x10^6^ cells harvested three independent experiments was purified using the Qiagen RNeasy Mini kit following manufacturer's instructions. RNA was quantified by Nanovue spectrophotometer. RNA quality was assessed on ExperionTM Automated Electrophoresis kindly provided by Dr. Martin Pfordt (Bio-Rad, Munich, Germany), and transcript levels were evaluated using the RT^2^ Profiler PCR array system (SA Biosciences, Germany).

### Reverse transcription

Total RNA (5 μg) was reverse transcribed using RT² First Strand Kit (SA Biosciences) according to the manufacturer’s protocol. Briefly, 2 μL genomic DNA elimination buffer (GE) were added to 5 μg total RNA (final volume 10μl) and incubated at 42°C for 5 min, chilled on ice for one min. and finally 10 μL RT-cocktail was added per reaction (4μl BC3: 5x RT buffer 3, 1μl P2 Primer, 2μl RE3: RT enzyme mix 3, 3 μl H_2_O). The mixture was incubated for 15 min at 42°C and finally at 95°C for 5 min, 91 μL H_2_O were added and the template cDNA was stored at -20°C for the subsequent use in the PCR array.

### RT² Profiler™ PCR Array

Real-time PCR was done by adding Template cDNA directly to PCR Master Mix containing RT^2^ Real-Time™ SYBR Green Master Mix and nuclease-free H_2_O (SA Bioscience). The mixtures were then aliquoted into 96-well PCR array plates (cDNA: 25 ng per 25 μL reaction), and thermal cycling was performed using a LightCylcer 480 system (Roche) applying the manufacturer's protocol as follows: 1 cycle at 95°C for 10 min. and 45 cycles: 15 sec at 95°C and 1 min. at 60°C. Each custom-designed plate permitted evaluation of 84 WNT-related transcripts plus controls. Data were analyzed using online software provided by the manufacturer (http://www.superarray.com/pcrarraydataanalysis.php). Fold up- and down- regulation of genes were calculated using the ΔΔCt method as described by the manufacturer (SA Bioscience). The genes have been normalized to the average expression of 2 housekeeping genes proven to be the most stable out of five examined genes (Beta-2-microglobulin, hypoxanthine phosphoribosyl transferase 1, ribosomal protein L13a, glyceraldehyde-3-phosphate dehydrogenase and β-actin) examined by the geNorm software (downloaded from www.gene-quantification.com). Only the genes that were up- or down-regulated by an average of ≥ 2-fold change are listed and discussed:

FRE; OS1; FZRB; hFIZ; FRITZ; FRP-3; FRZB1; SFRP3; SRFP3; FRZB-1; FRZB-PEN; FRZB.

### Statistical analysis

Wilcoxon’s rank-sum test was applied to evaluate the significance of comparing various groups to the controls, and PCR results were analyzed using pair-wise fixed reallocation randomization test using REST 2009 software (Qiagen). Significance was considered at p< 0.05. Otherwise, else indicated, all data indicate means±SD of at least three independent experiments.

## Results

### EP and EL inhibit proliferation and clonogenic growth of leukemia cells

**Fig 1:**
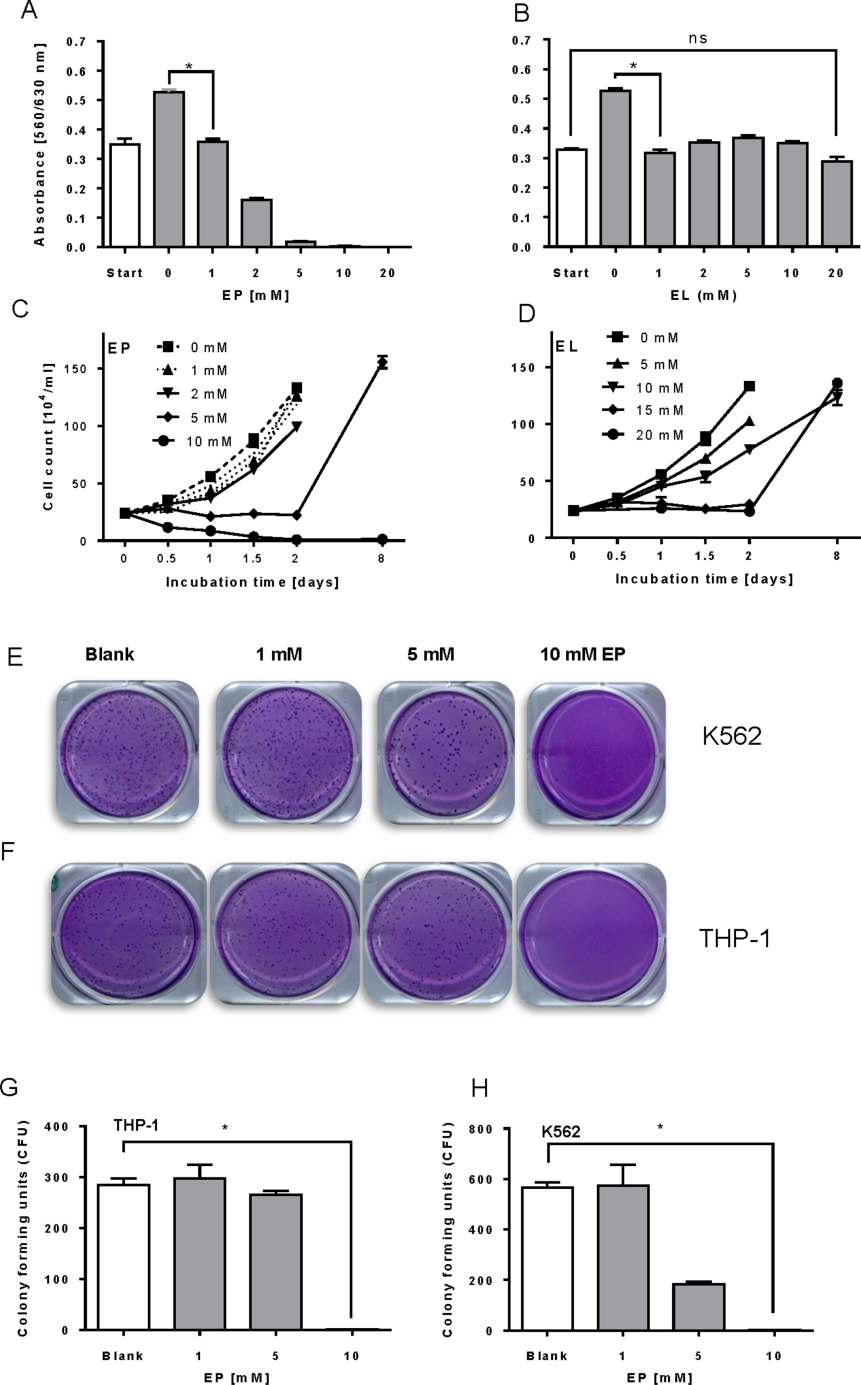
Growth inhibition of leukemia cells by EP and EL. (A, B) THP-1 cells (5000 cells/well) were seeded (start) and cultured at 37°C / 5% CO_2_ in the absence or presence of increasing concentrations of EP ort EL for 24 h. After that, cell proliferation was evaluated using the WST-1 assay. Data represent the mean ± SD of three independent experiments. (C, D) Reactivation studies show treatment of THP-1 cells by EP and EL for defined time intervals followed by medium replenishment. THP-1 cells (10^4^ cells/mL) were cultured in 75 ml-flasks in the absence and presence of graded concentrations of EP or EL for 24 h. After certain time points aliquots of cell suspension were removed for vitality testing by the trypan blue exclusion test. Reactivation of growth inhibition was analyzed by suspending cells at day 2 in fresh medium without inhibitors. The ordinate shows the number vital cells only. Data represent the mean ± SD (n = 3). (E-H) Colony formation assay: K562 and THP-1 cells (2.5 x 10^5^ each) were contacted with increasing concentrations of EP for 24 h in RPMI-FCS before seeded into a 0.3% agar layer of Petri-dishes in the absence of EP. Colony formation was check 14 days after incubation. (E) K562 cells; (F) THP-1 cells. (G, H) Statistic analysis of counted cell colonies (n = 3).

EP inhibited the proliferation of THP-1 cells in a dose-dependent manner as analyzed by the WST-1 proliferation/vitality assay. Cell proliferation was hampered by EP already at low concentrations but toxic effects were observed beyond the IC_50_ (1.4±0.2mM) discernible at lower O.D. values compared to controls (start) (Fig 1A). On the other hand, the reduced compound, EL impeded cell proliferation at a constant level, though it did not cause massive cell death like EP (Fig 1B).

Next, we examined the influence of an extended exposition of both compounds on vitality of THP-1 cells by applying graduated concentration of EP and EL for up to 48 hours (Fig 1C and 1D). At certain time points, cells were removed, and their vitality was inspected by trypan blue exclusion test, and then followed by complete medium refreshment. At low concentrations of EP (0–2 mM), cells continued to proliferate, whereas 5 mM EP arrested cell growth but cells retain their growth activity upon washing out EP from the medium. Interestingly, this recovery effect was absent in the presence of 10 mM EP, indicating an irreversible cell death. On the other hand, EL was found to reversibly arrest proliferation at 15 and 20 mM as cells recovered when it was omitted. This indicates that EL-treated cells were quiescent, temporally out of the proliferative cell cycle.

Colony formation in soft agar is considered an *in vitro* marker for tumorigenesis. As seen pre-treatment of cells with EP suppressed colony formation of THP-1 cells significantly (Fig 1F and 1G). Already at 10 mM EP, cancer cells lost their capability of forming cell colonies The durable effect of EP is based on the notice that the agar medium contained no EP thereafter, meaning that EP inhibited colony formation once the malignant tumor cells were in contact with the compound.

The study was extended to the human chronic myelogenous leukemia (CML) cell line K562. These cells are characterized by a 9;22 translocation resulting in the expression of the fusion oncoprotein, BCR/ABL. This oncoprotein exhibits a constitutively active kinase that confers the growth potential and apoptosis resistance of CML cells. We treated K562 cells with EP and found comparable sensitivity to this compound with respect to proliferation and colony forming capacity indicating common principle of action of EP in highly proliferative leukemia cells (Fig 1E and 1H).

### EP causes metabolic depletion of ATP in leukemia cells

**Fig 2:**
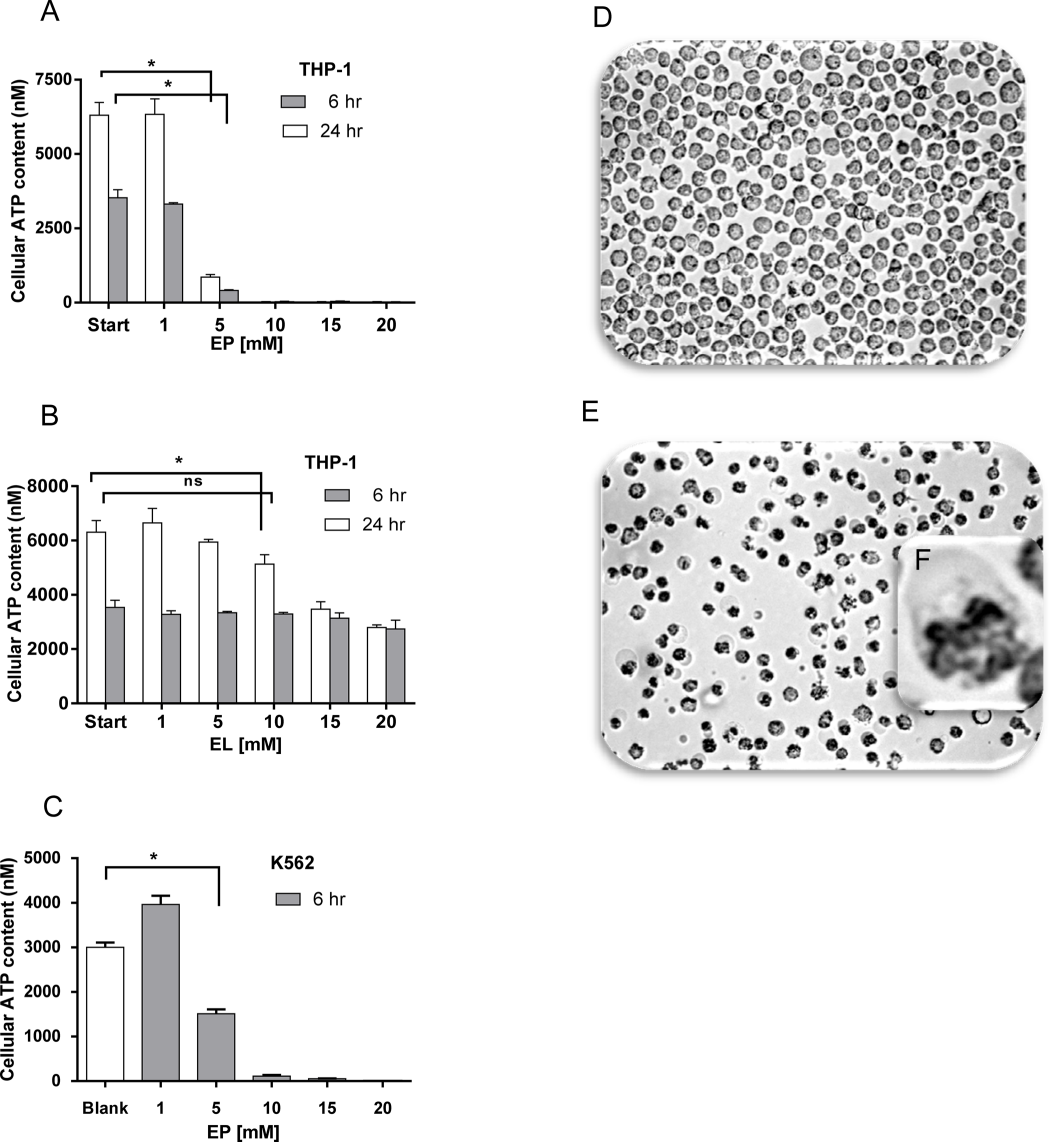
Effect of EP and EL at cellular ATP content of THP-1 and K562 cells. (A), THP-1 cells were seeded (5000 cells/well) and cultured in the presence of increasing concentrations of EP (0–20 M) and (B) EL (0–20 M) for 6h and 24 h, respectively. Cellular ATP content was determined using the CellTiter-Glo® Luminescent Cell Viability Assay. Data represent the mean ± SD of three independent experiments (n = 12). (C) Effect of EP on ATP content in K562 cells. Microscopic inspection of THP-1 cells grown in medium for 24 h in absence (D) or presence (E) of 10 mM EP (magnification: x20). (F) Enlargement of an aspect of *E*.

Growth arrest seen at low EP and higher EL concentration (Fig 1) is known to occur also under starvation condition that goes along with a cellular ATP deficiency. To check whether EP and EL affect metabolic activity of the tumor cells we analyzed the ATP content of THP-1 and K562 cells treated with various concentrations of EP and EL (Fig 2A–2C). We found that EP strongly decreased cellular ATP in a concentration-dependent manner yielding to an almost complete depletion at concentrations above 10 mM in both cell lines. The steep drop of ATP becomes already visible at 6 hours of incubation. By trend, EL disclosed comparable effects with the exception that the ATP level did not fall below the value of the untreated cells at short time incubation of 6 hours. EP-treated cells lost integrity, showed signs of increased cytoplasmic volume and destructed plasma membranes (Fig 2D–2F) while no massive cell death was observed in case of EL.

### Ethyl pyruvate interferes with glycolytic/para-glycolytic pathway in THP-1 cells

**Fig 3:**
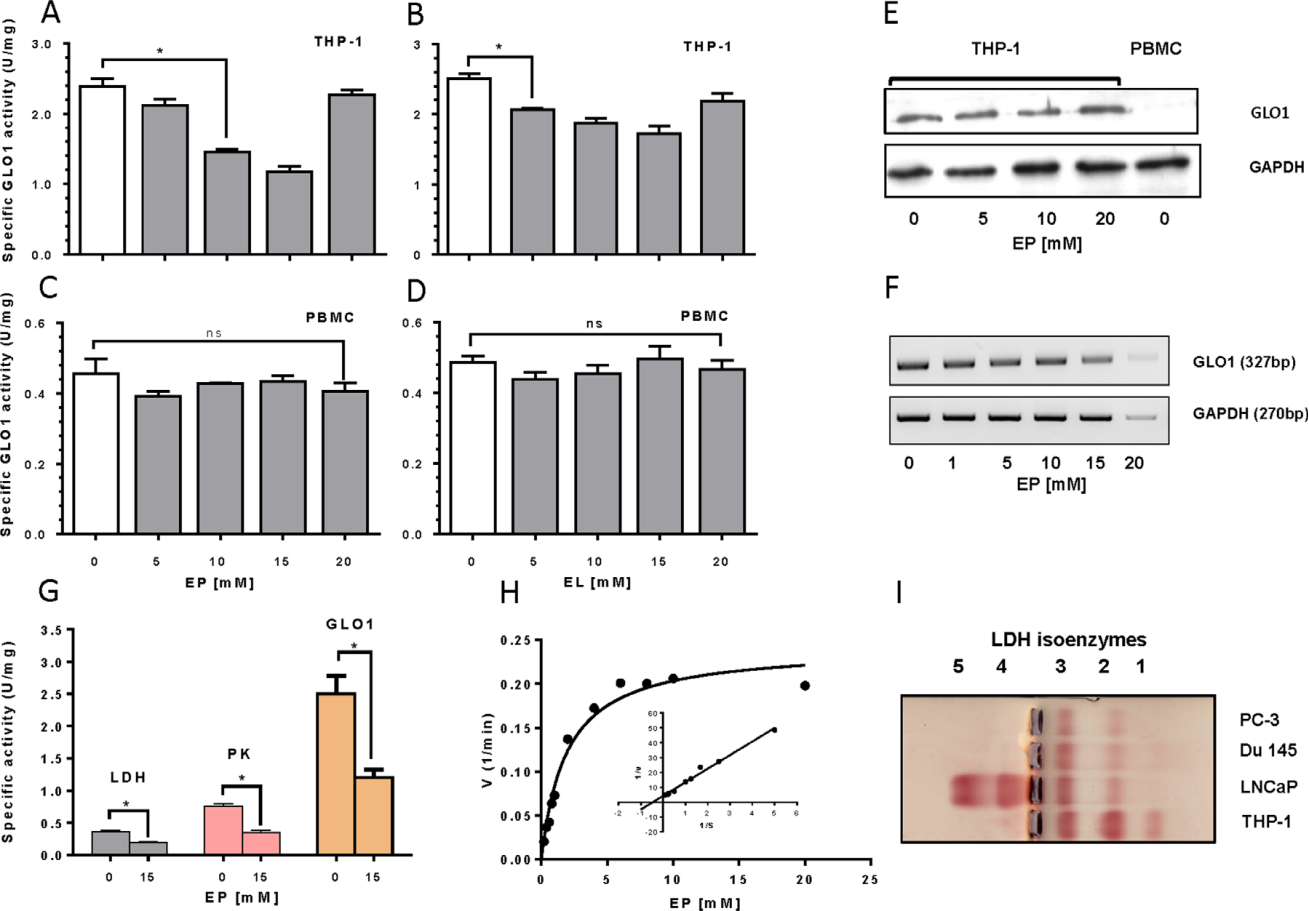
Analysis of glycolytic/paraglycolytic enzymes in THP-1 cells and PBMCs. (A-D) THP-1 cells and PBMCs were cultured RPMI 1640 medium containing 10% FCS in the presence of increasing concentrations of EP and EL for 24 h. Cell extracts were prepared from washed cells and analyzed for protein content and GLO1 activity. (E), Western blot analysis for GLO1 in THP1 cells treated with increasing concentrations of EP (0–20 mM). GLO1 in PBMC extract is shown for comparison. GAPDH was used as internal loading control. (F) Expression of GLO1-–mRNA in THP-1 cells treated with EP. (G) THP-1 cells were treated without and with 15 mM EP for 6 h followed by measurement of enzyme activity of GLO1, LDH and PK in the cytosolic extract. (H) V/S plot of activity of purified LDH-1 (SigmaAldrich, Germany) in dependence on increasing concentrations of EP (Michaelis-Menten-Plot); Insert depicts the Lineweaver-Burg-Plot. (I) LDH isoenzymes distribution in diverse tumor cell extracts as analyzed by agar gel electrophoresis.

It is known, that most tumor cells gain the energy from glycolysis rather than from oxidative phosphorylation. Therefore, to explain the detrimental effect of EP on leukemic cells we checked the expression and activity of glycolytic and para-glycolytic enzymes in THP-1 cells under EP treatment. We found that GLO1 is highly expressed in monocytic leukemia tumor cells in comparison to human PBMC as analyzed by enzyme activity measurement (Fig 3A–3D), Western blot (Fig 3E) and mRNA expression (Fig 3F). Treatment of cells with EP and EL revealed inhibitory effects on cytosolic GLO1 activity (Fig 3A–3D). In contrast, no significant analogous effect at GLO1 activity was observed in PBMCs incubated with EP and EL. The specific activity of the GLO1 in PBMC was found to be approximately 6 times lower compared to tumor cells. On the other hand, no effect of EP on GLO1 protein and mRNA level in THP-1 cells was observed with the exception for 20 mM EP (Fig 3E and 3F). Thus, EP inhibits GLO1 activity but does not affect its level of expression.

In addition to GLO1, we analyzed the impact of EP on hexokinase (HK), phosphofructokinase-1 (PFK), PK and LDH. While HK and PFK of THP-1 cells were not inhibited by EP, a significant inhibitory effect was ascertained for PK and LDH (Fig 3G). The observed inhibition of PK activity by EP was recently described and can best be explained by competing with binding of phosphoenolpyruvate to the active center of the enzyme [20]. Our data indicate that EP serves also as a substrate of LDH with a Km- and Vmax-values of 1.918 (±0.23) and 0.242 (±0.015), respectively (Fig 3H and insert). In addition, we found that the LDH-1 isoenzyme is dominantly expressed in THP-1 cells (Fig 3I). Thus, it is likely to assume, that the EP-induced depletion of the cellular ATP might be caused by a combined action at GLO1, LDH and PK [21].

### EP and EL induce apoptosis/necrosis in THP-1 cells but are less violate to human monocytes

**Fig 4:**
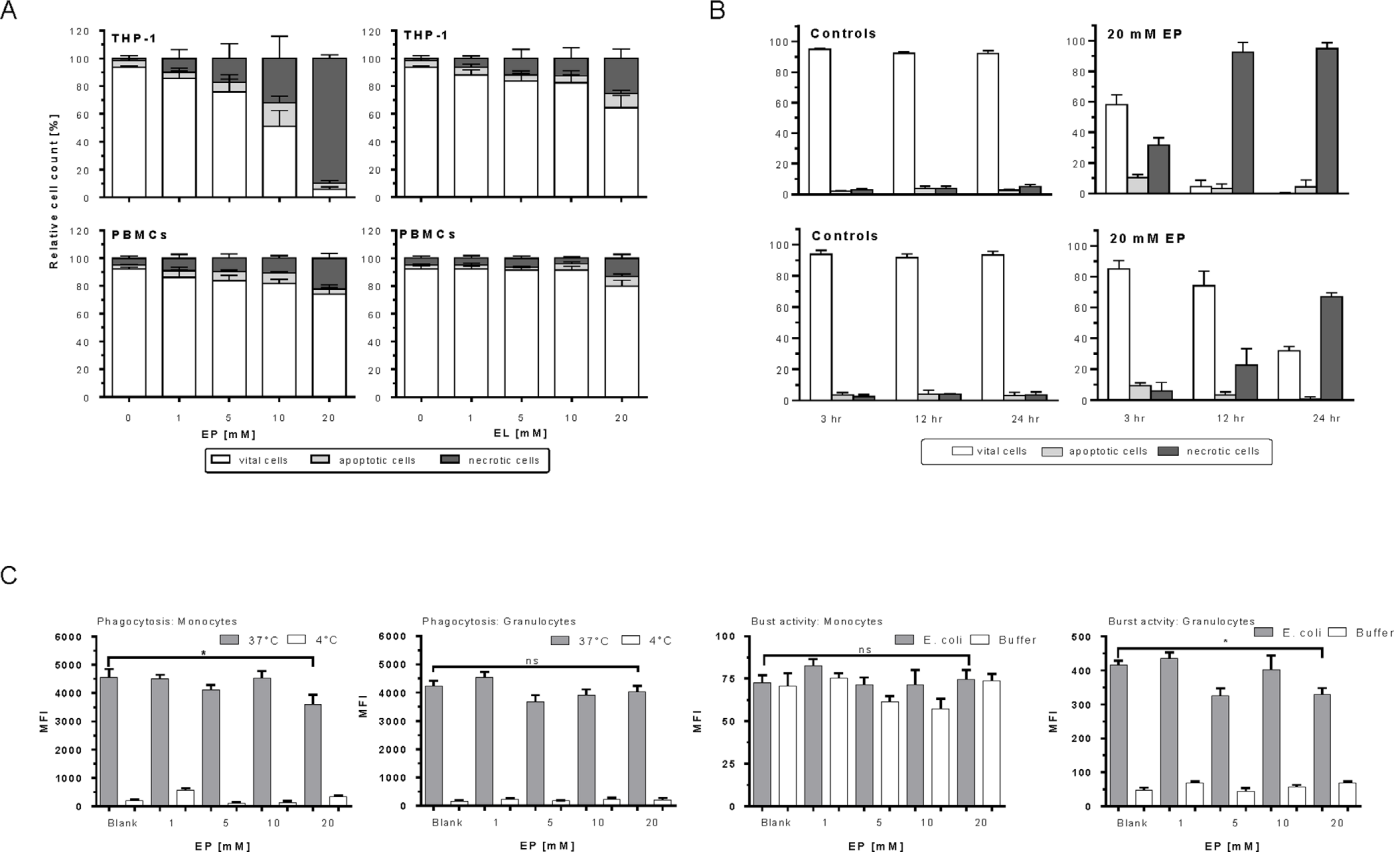
EP/EL induces necrosis/apoptosis of THP-1 cells but do not impair innate immune functions of normal white blood cells. (A) Flow cytometric analysis of THP-1 cells and blood monocytes upon treatment with EP and EL. THP-1 cells and blood monocytes were treated with EP and EL at increasing concentrations (1 to 20 mM) for 12 h at 37°C, then stained with annexin-V-FITC/PI, and apoptosis/necrosis was determined by flow cytometry. Results of four independent experiments are shown. The columns depict the mean percentage of vital cells, apoptotic cells, and necrotic/late apoptotic cells. Apoptotic cells are annexin-V-positive only, whereas necrotic cells are PI-positive and annexin-V-positive. (B) Time-dependent effects of EP on death induction of THP-1 cells and blood monocytes. THP-1 cells (upper row) and PBMCs (lower row) were treated with 20 mM EP for 3, 12 and 24 h and apoptosis/necrosis is measured. Controls represent untreated cells (n = 3). (C) Effect of EP at phagocytic and burst activity of blood cells. Heparinized human whole blood was incubated with increasing concentrations of EP at 37°C and 6 h, and a sample without stimulus served as negative background control. Blood was further incubated with fluorescein-labeled opsonized *E*. *coli* bacteria for 10 min at 4°C or 37°C, respectively, to measure phagocytic activity. Burst activity was analyzed by treatment of blood with 2 x 10^8^ opsonized, non-labelled E. coli for 10 min at 4°C and 37°C in the presence of dihydrorhodamine 123. The mean fluorescence intensity (MFI) of monocytes and granulocytes were identified by flow cytometry (n = 3).

To elucidate the mechanism of growth inhibition or cell death, we analyzed the degree of apoptosis/necrosis of treated THP-1 cells by flow cytometry using annexin-V-FITC and PI. EP strongly inhibited cell growth by inducing primarily cell necrosis in a concentration-dependent manner (Fig 4A). More than 95% of THP-1 cells rendered necrotic following 12 hours incubation with 20 mM of EP. Within the same interval, a higher number of vital cells were preserved (61.7%) in case of EL indicating its lesser toxicity effect on the tumor cells.

In contrast, significantly minimal effects of EP and EL were observed at cognate non-tumor mononuclear cells obtained from human blood (PBMC) that were similarly treated. The majority of monocytes (approximately 80%) remained vital even at 20 mM EP in comparison to leukemic cells. EL shows only marginally effects on vitality of blood monocytes at least up to a concentration of 10 mM.

Next, we inspected the time-dependence effect of 20 mM EP on apoptosis/necrosis of THP-1 cells by incubating cells for 3, 12, and 24 hours (Fig 4B). As expected, with increasing incubation time the dead cell counts was elevated. Already at short-time incubation of THP-1 cells with EP (3 hours) approximately 40% of cells died due to apoptosis/necrosis but only 17% of blood monocytes were affected. A large therapeutic window becomes obvious following 12 hours treatment with EP. Almost all leukemia cells were destroyed but only 30% of blood monocytes were affected. Even after 24 h of incubation, no vital THP-1 cells were left but still 30% of blood monocytes survived.

The finding that these compounds were almost innocuous to blood PBMCs relative to THP-1 cells forced us to analyze the *ex vivo* functional properties of EP and EL at the innate immune system such as phagocytosis and burst reaction (Fig 4C). Neither EP nor EL had the capability of deteriorating monocytes and granulocytes potency to phagocytize fluorescence-labeled bacteria. While EP slightly affected burst reactivity of granulocytes, no significant effect was seen with monocytes having a priori less burst reactivity compared to granulocytes. These results clearly document the drastic but desirable differential effects of EP on normal and malignant cells.

### EL affects cell cycle progression in THP-1 cells

**Fig 5:**
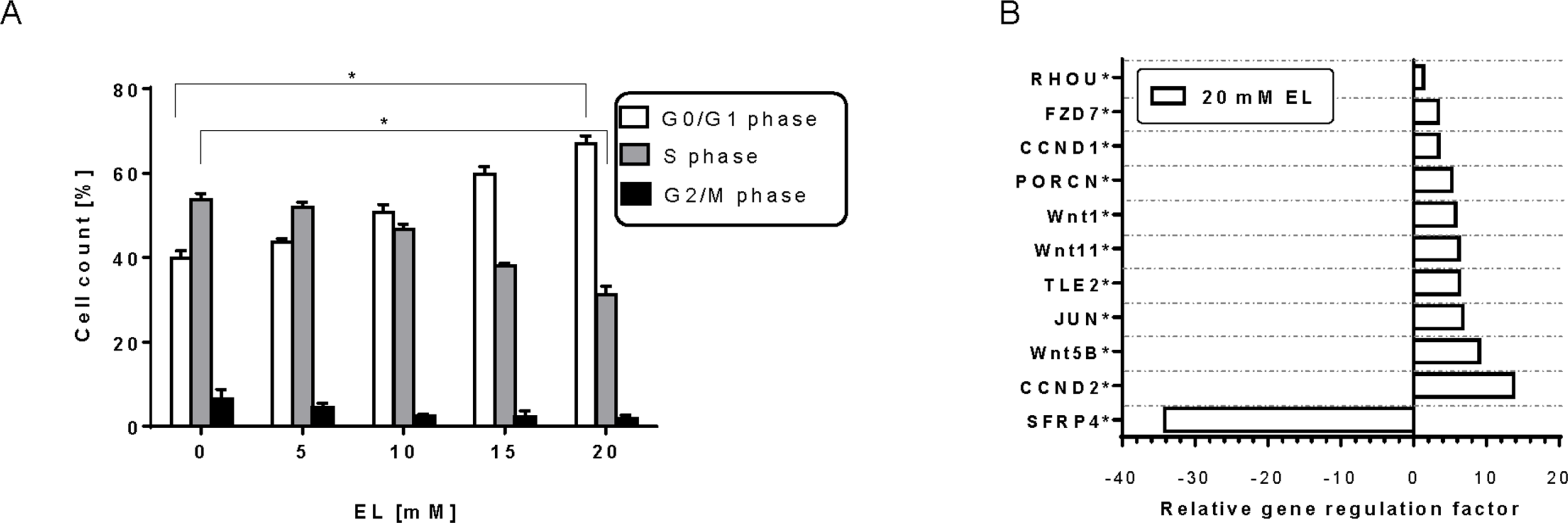
Impact of EL on cell cycle. (A) THP-1 cells were treated with EL for 24-h, stained by propidium iodine and subjected to cell cycle analysis by flow cytometry. All data represent mean ±SD. Statistical analyses were conducted among control (0 mM EL) and treated groups in G0/G1, S and G2/M phases separately, and different notations in the bar charts indicate statistical significance (n = 3). (B) THP-1 cells were exposed to 20mM EL for 24 h and mRNA expression was analyzed using a 96-well PCR-array of WNT-related transcripts. Data of 3 independent experiments demonstrated as the arithmetic mean of relative gene regulation factor. The asterisks represent significant differences relative to untreated controls (P was calculated in the Pair-Wise Fixed Reallocation Randomisation Test using REST 2009).*, P<0.05; **, P<0.01.

The signs of growth arrest as displayed in Fig 1 tell us that EL might exert its effect predominantly on cell cycle progression. Analyzing cell cycle by flow cytometry (Fig 5A) revealed that about 40% of the non-synchronized cells were in the G0/G1 phase, while 48% and 15% were in the S phase and G2/M phase, respectively. The percentage of cells in the G0/G1 phase increased dose-dependently up to 65% in the presence of 20 mM EL. This was accompanied by a reduced percentage of cells (31%) in the S phase (p> 0.01; n = 3 duplicates). These findings demonstrate that EL may block cell division by arresting THP-1 cells in the G0/G1 phase.

These results prompted us to analyze expression of genes involved in cell cycle regulation by using a PCR array of 84 WNT-related transcripts (Fig 5B). Because our interest was focused primarily at EL-mediated cell cycle arrest we incubated THP-1 cells with 20mM EL afore shown to arrest the tumor cells in the G0/G1 phase. Expression profile revealed significant up-regulation of 10 genes and down-regulation of SFRP4. Among them, 3 WNT-genes are up-regulated e.g. WNT 1, 11 and 5B as well as the frizzle receptor FZD7. Notable, the secreted frizzled-related protein 4 (SFRP4) is strongly down regulated by EL.

WNT target genes such as G1/S-specific cyclin-D1 (CCND1) and -D2 (CCND2) and the transcription factor AP1 (JUN) were also significantly up-regulated. The up-regulated PORCN gene product is involved in processing of WNT proteins by facilitating their palmitoylation and secretion.

These results demonstrate the overall tendency of EL to sustain cell integrity recognizable at the upregulation of WNT, FRZ- and LRP-mRNA that would turn out to facilitate WNT/β-catenin signaling, leading finally to sustainment of cell survival.

Because of the cell-disintegrating properties of EP observed already at 5 to 15 mM (see Fig 2) testing EP in the same way was needless.

### EP and EL inactivate GSK-3β by induced phosphorylation

**Fig 6:**
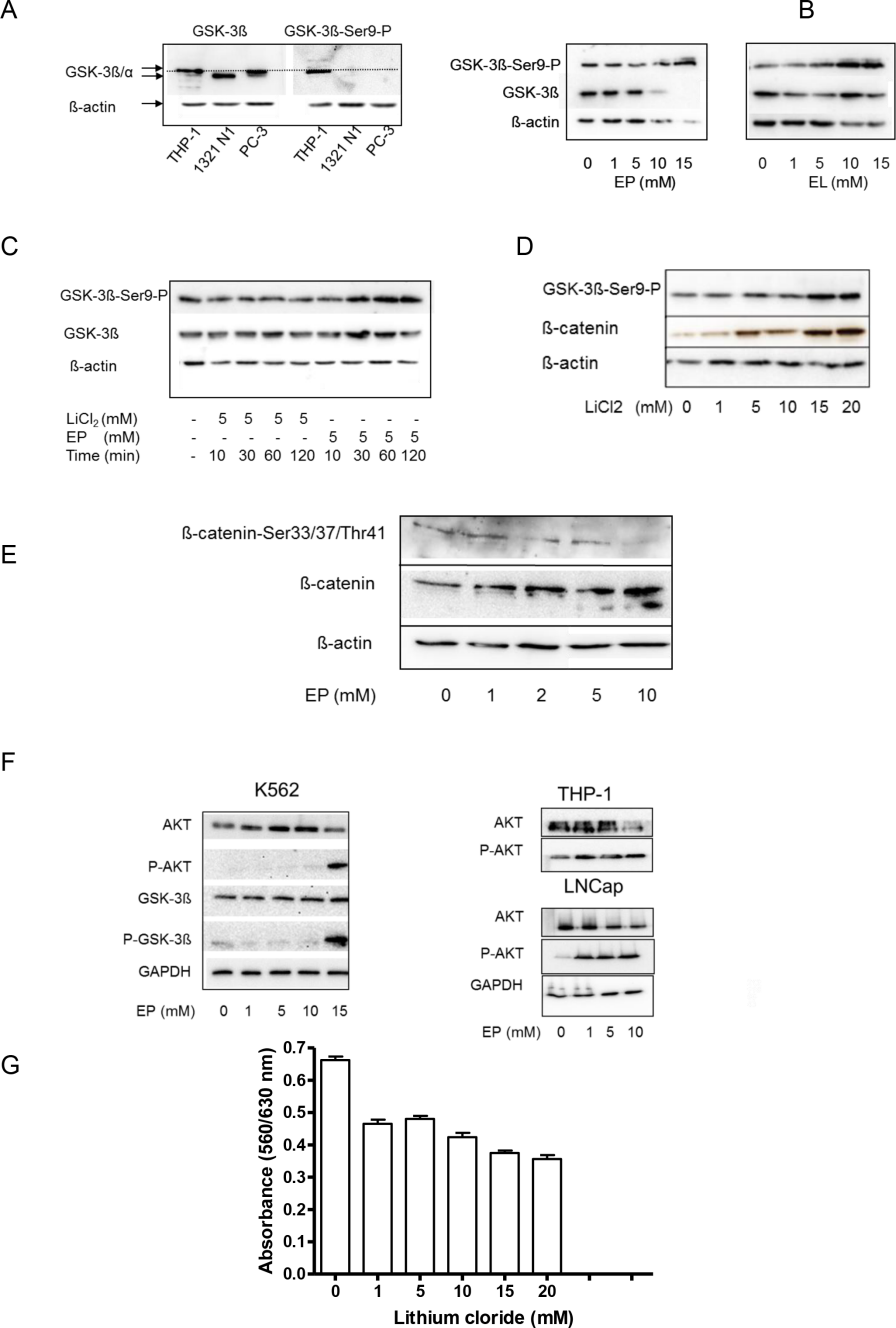
EP and EL inactivate GSK-3β by induced phosphorylation. Used Cell lines THP-1 (A-F), K562 (F),1321N1 (A), PC-3 (A) and LNCaP (F) were cultured at 37°C / 5% CO_2_ in the absence or presence of increasing concentrations of EP, EL or lithium for different times (10 min to 120 min (C) and 24 h (B, D-G), respectively. After that cells were harvested, lysed and subjected to immunoblots using specific antibodies against non-phosphorylated and phosphorylated proteins. Beta-actin and GPDH were used as loading control. (G) Cell proliferation of THP-1 cells in the presence of increasing concentration of lithium. Band intensities of selected blots were analysed by E.A.S.Y. Win 32 software and displayed in S Fig 1.

Results shown in Fig 5 allude to an active WNT/ß-catenin pathway in THP-1 cells. Therefore, we analyzed the expression of glycogen synthase kinase-3ß (GSK-3ß), which plays a central role within this signaling cascade [22]. In comparison to other tumor cells this enzyme has been found genuinely phosphorylated at Ser-9 in THP-1 cells leading finally to enzyme inactivation (Fig 6A). Results in Fig 6A also show that different tumor cells obviously express different GSK-3 isoforms (α- or β-form) that are encoded by distinct genes.

According to these data we hypothesized that the inhibitory effect of EP at cell proliferation might be due to inhibition of GSK-3ß phosphorylation. However, Western blot analysis of THP-1 cells incubated with increasing concentrations of EP showed opposite effects (Fig 6B). While the expression of GSK-3β decreased at higher concentrations of EP, the phosphorylation at Ser9 of the enzyme is augmented at the same time. EL followed the same trend but its effect on GSK-3β and β-actin was lower compared to EP.

Next, we studied the times course of EP´s action on GSK-3β compared to lithium (Fig 6C) Lithium is known to stimulate phosphorylation of GSK-3ß and thus enzyme inhibition [23]. The results reveal that EP stimulated phosphorylation of the enzyme significantly after a 120-min incubation period while no comparable effect was observed with lithium in this time range. This indicates a very early effect of EP at signal pathway members long before functional and morphological changes in THP-1 cells become apparent. In general, GSK-3β phosphorylation results in diminished phosphorylation of β-catenin. Therefore it should be expected that β-catenin degradation by the proteasome pathway is reduced. Indeed, EP as well lithium increase ß-catenin level in whole THP-1 cell extracts (Fig 6D and 6E). Results obtained by using a pan-phosphorylation site-specific antibody against β-catenin corroborate our interpretation that ß-catenin accumulation in the tumor cells might be due to the lack of phosphorylation, a known prerequisite for protein ubiquitination and proteasome degradation (Fig 6E).

The similarity between lithium and EP with respect to the active dose and induction of GSK-3ß phosphorylation is striking. However, results in Fig 6C clearly disclose higher phosphorylation-inducing activity of EP compared to lithium. In contrast to EP, lithium is not detrimental to THP-1 cells which resembles more the action of EL (Fig 6G). It is quite unlikely that EP induces auto-phosphorylation of GSK-3β, therefore we investigated upstream activators of GSK-3β such as Akt/PKB and found that EP also caused phosphorylation and thus activation of Akt/PKB (Fig 6F). Similar effects were observed with the leukemia cell line K562 and the prostate cancer cell line LNCaP.

## Discussion

### EP and energy metabolism

The cellular effect of EP has been mainly described as anti-oxidant [9] and anti-inflammatory [5, 21]. Recently, the effect of EP on tumor cells *in vitro* and *in vivo* has been described [7], however a currently acceptable molecular model of its action is missing.

We clearly showed here that EP is capable to inhibit the proliferation and tumorigenic properties of two human leukemic cell lines, the monocytic leukemic cell lineTHP-1, and the human chronic myelogenous leukemia (CML) cell line K562.

Our data indicate that the depletion of cellular ATP by EP is most probably the driving force for the death of leukemic cells as observed also by other tumor cells [24]. There are several mechanisms of cellular death: two such cell death pathways include apoptosis and necrosis, and recently the term necroptosis has been added [25]. While apoptosis is suggested to be ATP-dependent, necrotic cell death occurs under ATP-depleting conditions [26]. This could explain our notice of effective killing of THP-1 and K562 cells. As shown in Fig 4, the EP-induced cell death is mainly due to necrosis/apoptosis, with a negligible contribution of apoptosis. EP-treated cells show increasingly translucent cytoplasm, nuclear disintegration and increased cell volume (oncosis), culminating in the disruption of the plasma membrane, traits that remind to necroptosis [27] (Fig 2). Quite surprising is our finding that EP effectively perished leukemic cells and spared normal human blood cells including monocytes. Even functional important properties of white blood cells such as phagocytosis and burst reaction were not affected.

To elucidate the specific cellular targets of EP, we analysed at first the expression of GLO1 in THP-1 cells. This enzyme is highly expressed in these tumor cells when compared with their cognate blood cells, represented by the monocytes of PBMCs. Treatment of cells with EP inhibited GLO1 activity with a maximum between 10 and 15 mM, which is in the range of the IC_50_ for the isolated enzyme [21]. Inhibition of GLO1 terminates to accumulation of the reactive MGO, that is known to mediate its own harmful effects, amongst others, by induction of reactive oxygen species [28] and modification of macromolecules [29]. Mitochondrial proteins seem to be particularly prone to MGO-mediated post-translational modifications [30]. However, it is reasonable to assume that inhibition of GLO1 alone could not explain the EP-induced fast and almost complete impoverishment of THP-1 cells from ATP. Now, for the first time we disclosed interference of EP with the energy load of tumor cells and identified PK and LDH as the possible target. The inhibitory constant of EP for PK (K*i* = 3.0±0.29 mM) [20] is in the range of the concentration that causes most of its cellular effects. PK catalyses the last step in glycolysis converting the energy-rich substrate phosphoenolpyruvate (PEP) into pyruvate, while producing one molecule of ATP. One important key of controlling tumor growth lies therefore in the regulation of PK. Consequently, this would lead to a depletion of ATP (inhibition of PK), and accumulation of NADH (loss of pyruvate). The latter would either upstream block glycerol 3-P-dehydrogenase (for lack of proton acceptors) or being used as a co-substrate of LDH that can convert EP to EL (K*m* = 1.918 ±0.23). This concerted action would finally prevent oxidative phosphorylation of NADH in the mitochondria. Furthermore, the known inhibition of GLO1 by EP would lead to accumulation of the very reactive MGO exerting cell toxicity on its own. This interplay of substrate-enzyme relations could finally result in the observed loss of cellular ATP and thus cell death. Avowedly, the different cellular activity between EP and EL might also be a matter of concentration and penetration ability via transporters into the cells [31]. EL displayed only tendentious cellular effects because EL is not inhibitory to the purified enzymes neither to GLO1 nor to PK. Its impact can best be explained by the assumption that it acts as a prodrug being converted intracellular to EP by LDH or to lactate/pyruvate by nonspecific esterases [21]. The latter enzyme may produce pyruvate that was recently found to modulate cell fate at epigenetic level [32]. The selective killing of non-differentiated hematopoietic cells by EP may provide a means for the purging of leukemic cells from the peripheral bloodstream in patients undergoing autologous bone marrow transplantation. Thus, it can be expected that treatment of bone marrow aspirate with EP might be useful to destroy selectively tumor cells while leaving hematopoietic cell intact. The differential susceptibility of THP-1 tumor cells and its cognate blood monocytes toward EP awaits attention. It seems plausible to assume that highly proliferative cells like tumor cells possess a higher glycolytic flux that makes them more prone to inhibition of GLO1 and PK. The finding that most tumor cells harbour the highly active M2-PK isoenzyme that is absent on normal lymphatic cells sheds light on the differential effects observed.

Thus, the rate of metabolic activity of cells may make the difference in death-inducing activity of EP.

### EP, EL, lithium and GSK-3β

Surprisingly, we found that EL modulated the WNT/β-catenin pathway. Within this insulated cascade inhibition of GSK-3ß plays a central role and affects the half-life of 20% of all cellular proteins which leads to the assumption that WNT/β-catenin signalling controls global protein half-life [33].

Its role in cancer is quite differently discussed and may depend on its expression, state of phosphorylation as well as its interaction with other signalling networks. Inhibition of GSK-3β activity leads to stabilization and accumulation of β-catenin in the cytosol, which is shuttled into the nucleus and regulates gene expression of e.g. c-myc, c-jun and metalloproteases and of other pivotal cell factors [34,35]. We found that the GSK-3β is constitutively inhibited in THP-1 cells indicated by the presence of phosphorylated Ser9. This could be causative of the high proliferation rate of these leukemic cells on one site and its susceptibility to proliferation inhibitors on the other site. GSK-3β has been allocated a bi-functional role as a mediator of pro-survival signals and facilitator of apoptosis [34]. It was shown that GSK-3ß is involved in the apoptosis regulation of the NF-кB signaling pathway by facilitating NF-кB function [36] and inhibition of GSK-3β may abrogate this protective effect. Interestingly, also EP was recently considered to target NF-кB by modifying of specific cysteine and/or arginine residues causing inhibition of this pathway [37].

For the first time we show that GSK-3β is a target of EP by forcing Ser-9 phosphorylation. *In vivo* this modification is mediated by several kinases [34]. In addition, we found that EP can initiate the activation of upstream kinases like Akt/PKB or PI3-kinase leading downstream to phosphorylation of GSK-3β.

These findings however contradict its observed cankered effect on tumor cells. It is conceivable to assume that other mode of action such as ATP depletion may override its inhibitory effect on GSK-3ß allowing cell death-activating signals to be dominant. Lithium is a well-known specific and non-competitive inhibitor of GSK-3β *in vitro* and *in vivo*. Cumulative evidence has pointed to the potential of lithium as an anti-cancer agent. It has been shown to induce cell growth arrest, apoptosis, generation of ROS, and terminal differentiation in various human malignant tumors by targeting GSK-3β [34,35]. Comparing the phosphorylation of GSK-3β by EP and lithium, we found striking similarities with respect to the effective concentration as well as the targets. However, it has to be stated that the phosphorylation induced by EP occurs much faster when studied in a cell-based assay indicating the involvement of additional and probably different mechanisms of action. Whether EP affects the phosphorylation of GSK-3β by its capability to complex divalent cations, e.g. Mg^2+^ or Zn^2+^, needs further investigation [5,38]. Our report is also consistent with the previous findings of others who have shown that GSK-3β inhibitors are capable of suppressing the growth of a number of tumors like leukaemia, glioblastoma, breast and colorectal cancer [39]. For instance, treatment of cells with BIO, a potent GSK-3β inhibitor, suppressed cell growth, induced apoptosis in leukemic cells and delayed tumor formation in a mouse model of leukemia [40]. This is striking, because similar to our notice the authors found strong β-catenin accumulation in treated cells accompanied by cessation of the up-regulation of β-catenin target genes including TCF-4. BIO treatment specifically killed leukemic cells and spared normal stem cell [41]. Surprisingly, we found a similar distinct action of EP on leukemic cells and normal blood cells.

**Fig 7:**
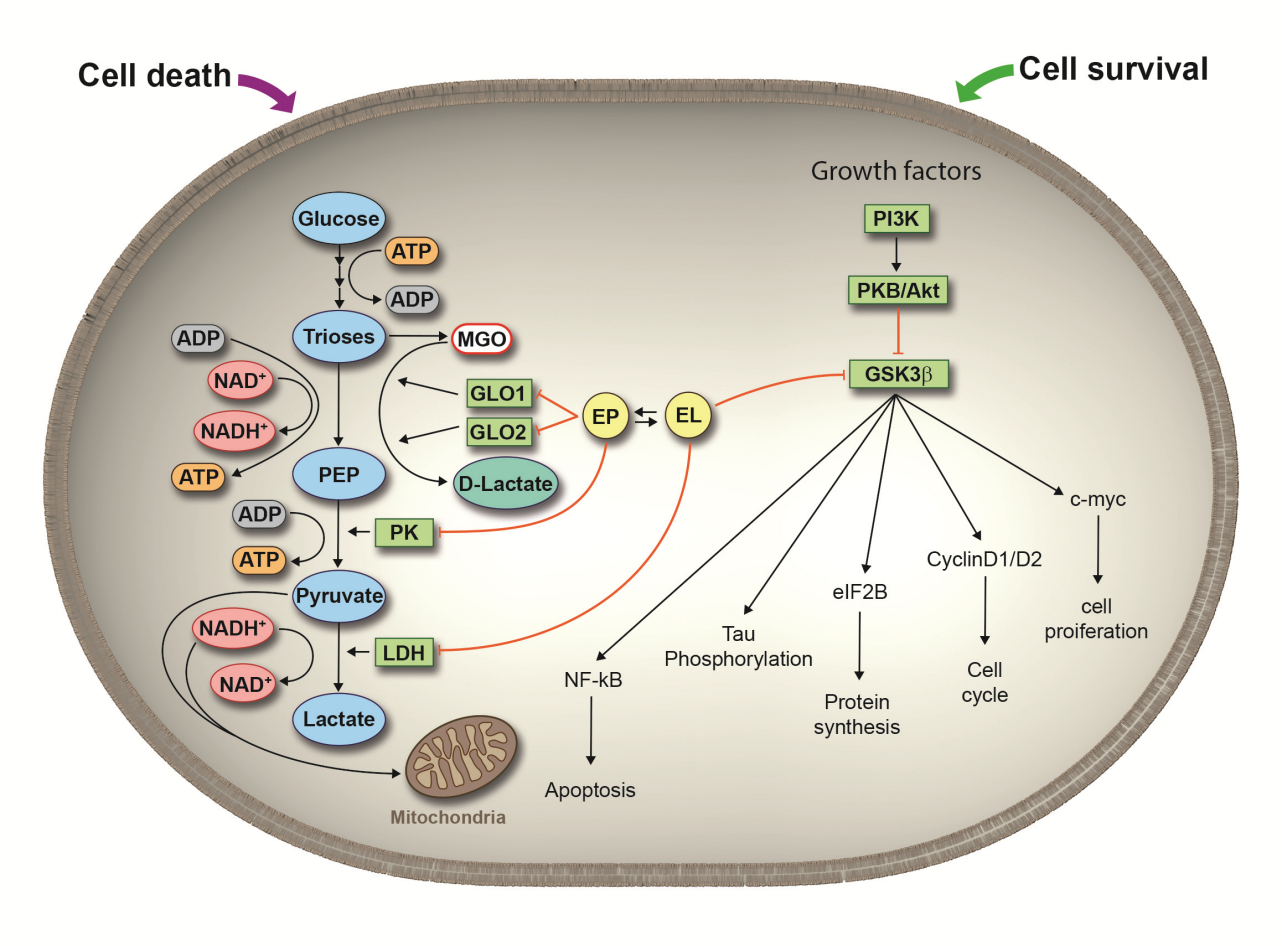
Dual regulation of cell death and survival by EP and EL. Induction of cell death by necrosis/apoptosis in tumor cells exhibiting a high glycolytic throughput is primary mediated by depletion of ATP due to inhibition of PK and LDH by EP while inhibition of GLO1/GLO2 yields to accumulation of the cell toxic MGO. EL is not inhibitory to these enzymes and therefore not toxic to cells. EL predominantly stimulates pivotal cell pathways primarily through induction of phosphorylation of GSK-3ß. Which mechanism may dominate depends on the degree of glycolysis, expression of monocarboxylate transporters (MCT) for EP/EL and the type of LDH isoenzyme in the cells.

In contrast to EP, EL induced cell cycle arrest in the G0/G1 phase (Fig 5). We checked the involvement of p53, but we did not find detectable immunoreactive p53 protein. This recalls the assumption that THP-1 cells possess mutated inactive p53 [42], which means that the cell cycle arrest must be caused by a p53-independent mechanism. It is known that starvation may arrest cells in G1/G0 which is determined by the energy load of the cells. We assume that the action of EL is probably due to a graduated depletion of cellular ATP (Fig 2). Overall, EL tentatively inhibits cell proliferation by blocking cell cycle progression. However, the induction of members of the cyclin D family, CCND1 and CCND2, WNT family members as well as the proto-oncogene JUN reminds more to pivotal effects of EL. This is confirmed by the finding that inhibition of GSK-3ß leads to stabilization of cyclin D1 [43]. Furthermore, SFRP4 is known to inhibit β-catenin dependent WNT/ß-catenin signaling and decreased transcription of WNT/ß-catenin target genes, Axin2, CyclinD1 and Myc [44]. Inhibition of SFRP4 indicates abrogation of its inhibitory effect on ß-catenin dependent WNT/ß-catenin signaling thus resulting also in pivotal effects. Overall, EL seems to activate survival mechanisms in response to stress signals. The proposed mechanism of action of EP and EL in THP-1 cells is illustrated in Fig 7.

Apart from tumors, GSK-3β has been implicated in the progression of multiple human conditions including Alzheimer`s disease, bipolar disorder and noninsulin-dependent diabetes mellitus [45, 46]. In Alzheimer´s disease, active GSK-3ß leads to hyper-phosphorylation of Tau probably due to lack of Akt/PKB activity transducing an increase in GSK-3β activity [47]. Involvement of GSK-3β in psychotic disorders is already evidenced and mood stabilizers have been shown to modulate GSK-3β activity [48]. Lithium has been an FDA-approved drug for the treatment of mood disorders but new inhibitors of GSK-3ß appear to be valuable for treatment of different neurological pathologies. Thus, the use of EL (preferred to EP) for treatment of brain diseases might be anticipated due to its inhibitory effect on GSK-3β and its pivotal impact at cellular level. Studies documenting protective neurological effects as well as the preservation of gut mucosal and brain barrier functions might be connected to this novel property of EP/EL [11–13].

## Supporting Information

S1 Fig Fig 6 Quantification of Western blots. Relative band intensities of selected Western blots shown in Fig 6 were calculated as the ratio of the protein band intensity/ß-catenin band intensity using the E.A.S.Y. Win 32 software. Data were presented as mean±SD (n = 3); (*) indicates a P-value<0.0.5.
(PDF)
