# Supplementary figures and images for: Ethyl Pyruvate Combats Human Leukemia Cells but Spares Normal Blood Cells
Source: PLoS One. 2016 Aug 31;11(8):e0161571. doi: 10.1371/journal.pone.0161571 (PMC5006986; doi:10.1371/journal.pone.0161571)

## Supplementary Information

S1-Fig 6B

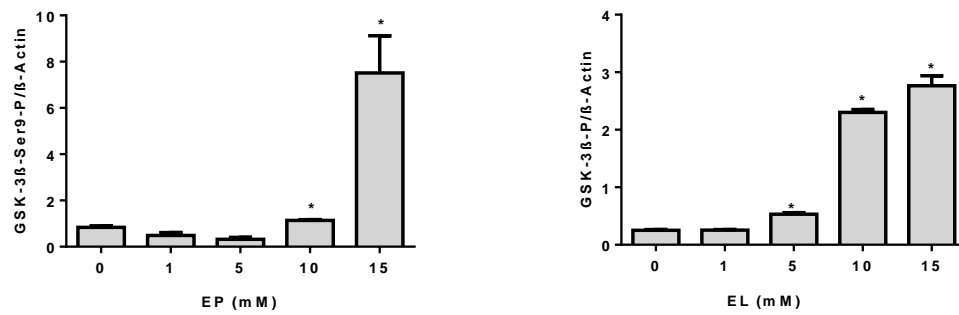

S1-Fig 6C

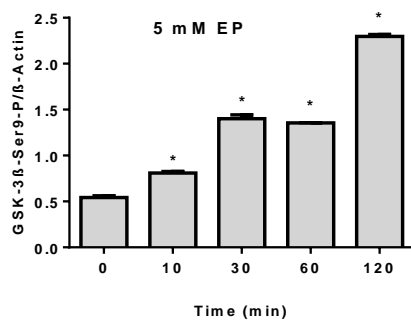

S1-Fig 6D

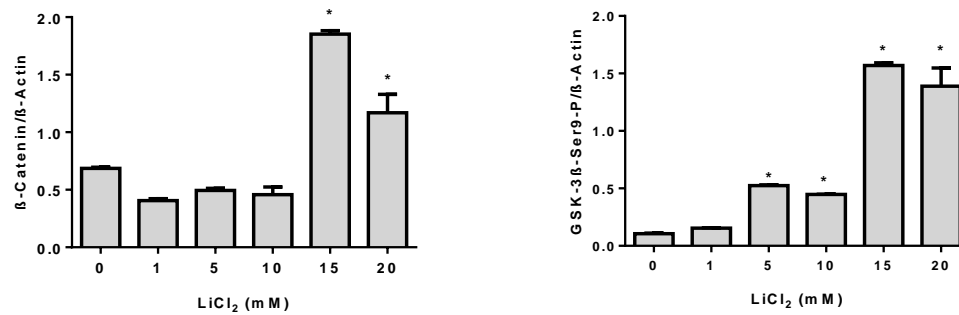

S1-Fig 6E

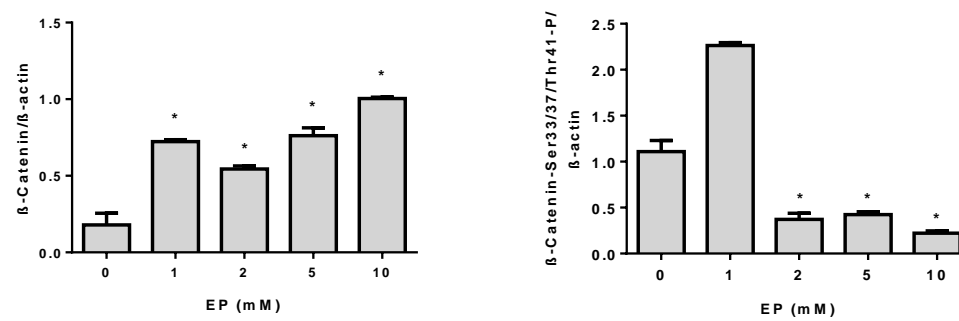

Supplement: S1 Fig — Relative band intensities of selected Western blots shown in Fig 6 were calculated as the ratio of the protein band intensity/ß-catenin band intensity using the E.A.S.Y. Win 32 software. Data were presented as mean±SD (n = 3); (*) indicates a P-value<0.0.5. (PDF) [file pone.0161571.s001.pdf]
